# Supplementary material for: Altered microRNA Transcriptome in Cultured Human Liver Cells upon Infection with Ebola Virus
Source: Int J Mol Sci. 2021 Apr 6;22(7):3792. doi: 10.3390/ijms22073792 (PMC8038836; doi:10.3390/ijms22073792)
Supplement: Supplementary file 1 [file ijms-22-03792-s001.zip › Supplementary_File/C_ GO_Analysis_Results/16-30nt_go_Reston-96h-Huh7_vs_Control-96h-Huh7_down.mature_mirna_targets/MF_result(Human).html]

| GO.ID | Term | Ontology | Count | Pop.Hits | List.Total | Pop.Total | Fold.Enrichment | Pvalue | FDR | Enrichment.Score | Gene.Ratio | GENES |
| --- | --- | --- | --- | --- | --- | --- | --- | --- | --- | --- | --- | --- |
| GO:0005515 | protein binding | Molecular function | 275 | 11565 | 370 | 17548 | 1.1277503184118 | 0.000260992314693599 | 0.338246039842904 | 3.58337228091958 | 0.743243243243243 | CPLX1//LRRK2//VAMP3//GOSR2//ELK1//BHLHE40//GNAO1//GNAZ//PROK1//NUCB1//RIC8B//HIF1A//MAEA//PARVA//EPS8L2//MLPH//DIXDC1//MICAL2//NOS3//SKAP1//PARD6B//RALGPS2//ARHGEF6//GRIN2B//NCAM1//TRDN//CADM2//TNFSF4//TNFSF14//NDP//SFRP1//EBI3//GDF9//BMP10//IFNK//FAM83B//ITGA5//RASL11B//NISCH//EMP2//TLN1//JAM3//FERMT3//TG//CTH//EWSR1//PPP3CA//PPP3CB//KCNQ4//HTRA4//CXCL11//PTPRT//CTNND2//SKP1//KIF2A//MAPRE1//RBFOX1//PRKAA1//YWHAB//FGF11//KRT2//EPAS1//HEYL//INSIG2//PURA//YWHAZ//PAXBP1//LARP1//RHOH//SH3BP5//TBC1D22B//FZD2//TRABD2B//FZD3//SMAD1//RALBP1//UBE4B//COPS5//KMT2E//PEX5//TNKS2//TSPAN14//TTC28//CDKN1B//RHOF//MAPK4//CD8A//BAG5//CARHSP1//SH3YL1//PPP1R26//PPP1R3F//ARFIP2//MLF1//PRKAR2B//SYT14//ABCC8//DBI//CXXC4//SEMA4G//DNAJB12//TOMM34//HSPA13//RNF217//SLC25A5//RTN4//AGTR1//PALM//SEPT7//UBXN2A//VLDLR//IGSF1//FOXP3//SPIN3//ASF1B//DLAT//ALAD//P2RX2//SULT4A1//PDIA3//MCIDAS//CLDN18//KLHL7//CREBZF//RPE//SNRPA//CNTN2//BEST1//PIP4K2C//RNF135//BAG2//OXSR1//CALCOCO2//CCDC103//AADAT//PSMD7//TTK//CREB3L3//AOC3//NFS1//BEX4//UBXN2B//TP53INP2//BUB3//PFDN1//LRRC38//KCNIP3//KCNIP2//SCN3B//EIF4G2//CD46//UBFD1//SKIL//EFNA3//SIM1//ANO3//BRK1//TNFRSF8//NDFIP1//ACTR3//ACTR2//AKAP10//SHQ1//DPYS//RNF169//TOMM40L//DLX5//CAPN7//PRDM4//PRDM12//CD81//TIMM23//PYURF//EMC8//PPIH//ZBTB6//PNRC1//FERMT2//CHRNA3//AP1S1//SLAIN1//UBXN10//LYPD6//PACRGL//LSM11//NEK7//USP54//SLC35G1//C18ORF54//SNX31//FAM9A//FAM9C//EIF4A2//FBXO45//HIPK1//FABP2//CPEB3//FOXG1//PAXIP1//RRP1B//ZBTB43//FAM168A//MGRN1//ATP1B4//LDOC1//NAT9//FBXL3//MYCBP//PTPN18//GPR3//WDR91//NRBF2//CDR2L//HMGB3//HMGN2//HOXC9//IL4R//FAM111B//C16ORF87//MEA1//MRC1//NRAS//PAX5//PLLP//RLIM//UCHL5//PMAIP1//POLR2E//USP53//PPP1R2//ENOX1//TMEM248//NUDT15//VPS53//RFK//DRAM1//STRBP//THUMPD1//MAP7D1//ZFP64//FAM214A//GRIPAP1//PTBP1//WDFY1//PCTP//RBM3//RRAS//BFSP1//CEP85//SMPD1//STATH//TMPRSS2//U2AF1//UBE2D3//UBE2E1//ZNF124//PAX8//RPAP3//NUP214//TXNDC5//TRIM7//CAMLG//CASP7//OGT//CGNL1//PARG//CYP4F2//STON2//ACTL6A//KBTBD6//SESTD1//NREP//COPS2//SCAMP1//CREB5// |
| GO:0016709 | oxidoreductase activity, acting on paired donors, with incorporation or reduction of molecular oxygen, NAD(P)H as one donor, and incorporation of one atom of oxygen | Molecular function | 5 | 44 | 370 | 17548 | 5.38943488943489 | 0.00224204335861499 | 0.842707519890619 | 2.64935599288951 | 0.0135135135135135 | CH25H//NOS3//CYP4F3//CYP4F2//MICAL2// |
| GO:0005048 | signal sequence binding | Molecular function | 5 | 47 | 370 | 17548 | 5.04542840713054 | 0.00300805080739185 | 0.842707519890619 | 2.52171483258054 | 0.0135135135135135 | PEX5//NUP214//TOMM40L//KDELR3//KCNIP2// |
| GO:0044325 | ion channel binding | Molecular function | 8 | 119 | 370 | 17548 | 3.18837156484215 | 0.00373074696904915 | 0.842707519890619 | 2.4282042051731 | 0.0216216216216216 | TRDN//LRRK2//LRRC38//KCNIP3//KCNIP2//SCN3B//ABCC8//YWHAZ// |
| GO:0050839 | cell adhesion molecule binding | Molecular function | 20 | 489 | 370 | 17548 | 1.93975570662687 | 0.00381536413522225 | 0.842707519890619 | 2.41846400702208 | 0.0540540540540541 | NISCH//EMP2//ITGA5//TLN1//JAM3//FERMT3//PTPRT//EIF4G2//MAPRE1//LARP1//ARFIP2//CD46//PARVA//UBFD1//RTN4//EPS8L2//YWHAB//YWHAZ//SEPT7//CADM2// |
| GO:0017147 | Wnt-protein binding | Molecular function | 4 | 31 | 370 | 17548 | 6.11961639058413 | 0.00390142370319731 | 0.842707519890619 | 2.40877688179071 | 0.0108108108108108 | FZD2//TRABD2B//SFRP1//FZD3// |
| GO:0035091 | phosphatidylinositol binding | Molecular function | 12 | 237 | 370 | 17548 | 2.40136845706466 | 0.00461557291196216 | 0.854540356271851 | 2.33577438411562 | 0.0324324324324324 | WDFY1//MARK1//SESTD1//FERMT2//OGT//ARFIP2//NISCH//SNX31//SH3YL1//TNFAIP8L3//TULP4//TLN1// |
| GO:0045296 | cadherin binding | Molecular function | 14 | 324 | 370 | 17548 | 2.04931598264932 | 0.00915024369324035 | 1 | 2.0385673394614 | 0.0378378378378378 | PTPRT//EIF4G2//MAPRE1//LARP1//ARFIP2//CD46//PARVA//UBFD1//RTN4//EPS8L2//TLN1//YWHAB//YWHAZ//SEPT7// |
| GO:0001614 | purinergic nucleotide receptor activity | Molecular function | 3 | 21 | 370 | 17548 | 6.77528957528958 | 0.00932839081311597 | 1 | 2.03019326742808 | 0.00810810810810811 | GPR34//P2RY14//P2RX2// |
| GO:0016502 | nucleotide receptor activity | Molecular function | 3 | 21 | 370 | 17548 | 6.77528957528958 | 0.00932839081311597 | 1 | 2.03019326742808 | 0.00810810810810811 | P2RX2//GPR34//P2RY14// |
| GO:0017075 | syntaxin-1 binding | Molecular function | 3 | 22 | 370 | 17548 | 6.46732186732187 | 0.0106350471291186 | 1 | 1.97326058118478 | 0.00810810810810811 | CPLX1//LRRK2//VAMP3// |
| GO:0042813 | Wnt-activated receptor activity | Molecular function | 3 | 22 | 370 | 17548 | 6.46732186732187 | 0.0106350471291186 | 1 | 1.97326058118478 | 0.00810810810810811 | FZD2//SFRP1//FZD3// |
| GO:0022839 | ion gated channel activity | Molecular function | 14 | 335 | 370 | 17548 | 1.98202501008471 | 0.0120061456414145 | 1 | 1.9205963927077 | 0.0378378378378378 | P2RX2//GRIN2B//ANO3//BEST1//CHRNA3//KCNIP3//SCN3B//SCN1A//SCN7A//LRRC38//KCNQ4//KCNIP2//ABCC8//JPH1// |
| GO:0005248 | voltage-gated sodium channel activity | Molecular function | 3 | 23 | 370 | 17548 | 6.186133960047 | 0.0120422710991585 | 1 | 1.91929160005336 | 0.00810810810810811 | SCN3B//SCN1A//SCN7A// |
| GO:0022836 | gated channel activity | Molecular function | 14 | 338 | 370 | 17548 | 1.96443307212538 | 0.0128943268992239 | 1 | 1.88960132367591 | 0.0378378378378378 | P2RX2//GRIN2B//ANO3//BEST1//CHRNA3//KCNIP3//SCN3B//SCN1A//SCN7A//LRRC38//KCNQ4//KCNIP2//ABCC8//JPH1// |
| GO:0035586 | purinergic receptor activity | Molecular function | 3 | 25 | 370 | 17548 | 5.69124324324324 | 0.0151629139103867 | 1 | 1.81921733078862 | 0.00810810810810811 | P2RX2//GPR34//P2RY14// |
| GO:0005072 | transforming growth factor beta receptor, cytoplasmic mediator activity | Molecular function | 2 | 10 | 370 | 17548 | 9.48540540540541 | 0.0178397165745119 | 1 | 1.74861204968542 | 0.00540540540540541 | SMAD1//CDKN1B// |
| GO:0016018 | cyclosporin A binding | Molecular function | 2 | 10 | 370 | 17548 | 9.48540540540541 | 0.0178397165745119 | 1 | 1.74861204968542 | 0.00540540540540541 | PPIH//PPP3CA// |
| GO:0090079 | translation regulator activity, nucleic acid binding | Molecular function | 3 | 27 | 370 | 17548 | 5.26966966966967 | 0.018697182389617 | 1 | 1.72822383543349 | 0.00810810810810811 | CPEB3//PURA//LARP1// |
| GO:0001618 | virus receptor activity | Molecular function | 5 | 74 | 370 | 17548 | 3.2045288531775 | 0.0199644295351845 | 1 | 1.69974309484911 | 0.0135135135135135 | ITGA5//CD46//MRC1//NCAM1//CD81// |
| GO:0104005 | hijacked molecular function | Molecular function | 5 | 74 | 370 | 17548 | 3.2045288531775 | 0.0199644295351845 | 1 | 1.69974309484911 | 0.0135135135135135 | ITGA5//CD46//MRC1//NCAM1//CD81// |
| GO:0000774 | adenyl-nucleotide exchange factor activity | Molecular function | 2 | 11 | 370 | 17548 | 8.62309582309582 | 0.0215034912260706 | 1 | 1.66749102393895 | 0.00540540540540541 | BAG5//BAG2// |
| GO:0035035 | histone acetyltransferase binding | Molecular function | 3 | 29 | 370 | 17548 | 4.90624417520969 | 0.0226487888866648 | 1 | 1.64495501633994 | 0.00810810810810811 | EPAS1//HIF1A//FOXP3// |
| GO:0005244 | voltage-gated ion channel activity | Molecular function | 9 | 198 | 370 | 17548 | 2.15577395577396 | 0.0248805210539614 | 1 | 1.60414052878465 | 0.0243243243243243 | GRIN2B//LRRC38//KCNQ4//KCNIP2//ABCC8//KCNIP3//SCN3B//SCN1A//SCN7A// |
| GO:0022832 | voltage-gated channel activity | Molecular function | 9 | 198 | 370 | 17548 | 2.15577395577396 | 0.0248805210539614 | 1 | 1.60414052878465 | 0.0243243243243243 | GRIN2B//KCNIP3//SCN3B//SCN1A//SCN7A//LRRC38//KCNQ4//KCNIP2//ABCC8// |
| GO:0050321 | tau-protein kinase activity | Molecular function | 2 | 13 | 370 | 17548 | 7.2964656964657 | 0.0296630188359943 | 1 | 1.52778465246146 | 0.00540540540540541 | MARK1//PRKAA1// |
| GO:0017048 | Rho GTPase binding | Molecular function | 8 | 173 | 370 | 17548 | 2.19315731916888 | 0.0305720408020915 | 1 | 1.51467556946809 | 0.0216216216216216 | EPS8L2//ARHGEF6//RALBP1//ARFIP2//BRK1//LRRK2//RHOH//PARD6B// |
| GO:0000900 | translation repressor activity, mRNA regulatory element binding | Molecular function | 2 | 14 | 370 | 17548 | 6.77528957528958 | 0.034132319883642 | 1 | 1.46683419286919 | 0.00540540540540541 | CPEB3//PURA// |
| GO:0001608 | G-protein coupled nucleotide receptor activity | Molecular function | 2 | 14 | 370 | 17548 | 6.77528957528958 | 0.034132319883642 | 1 | 1.46683419286919 | 0.00540540540540541 | GPR34//P2RY14// |
| GO:0045028 | G-protein coupled purinergic nucleotide receptor activity | Molecular function | 2 | 14 | 370 | 17548 | 6.77528957528958 | 0.034132319883642 | 1 | 1.46683419286919 | 0.00540540540540541 | GPR34//P2RY14// |
| GO:0005216 | ion channel activity | Molecular function | 15 | 424 | 370 | 17548 | 1.6778429372769 | 0.0360056169188934 | 1 | 1.44362974349392 | 0.0405405405405405 | P2RX2//GRIN2B//ANO3//BEST1//CHRNA3//SCN3B//SCN1A//SCN7A//LRRC38//KCNQ4//KCNIP2//SLC26A1//KCNIP3//ABCC8//JPH1// |
| GO:0005261 | cation channel activity | Molecular function | 12 | 316 | 370 | 17548 | 1.80102634279849 | 0.0364608116434116 | 1 | 1.43817366787162 | 0.0324324324324324 | P2RX2//GRIN2B//SCN3B//SCN1A//SCN7A//LRRC38//KCNQ4//KCNIP2//KCNIP3//ABCC8//JPH1//CHRNA3// |
| GO:0015267 | channel activity | Molecular function | 16 | 463 | 370 | 17548 | 1.63894693829899 | 0.037190626101513 | 1 | 1.42956651026315 | 0.0432432432432432 | P2RX2//GRIN2B//ANO3//BEST1//CHRNA3//KCNIP3//SCN3B//SCN1A//SCN7A//LRRC38//KCNQ4//KCNIP2//SLC26A1//ABCC8//JPH1//TOMM40L// |
| GO:0016597 | amino acid binding | Molecular function | 4 | 60 | 370 | 17548 | 3.1618018018018 | 0.0376544135635386 | 1 | 1.42418411178863 | 0.0108108108108108 | GRIN2B//NOS3//DPYS//DDAH1// |
| GO:0022803 | passive transmembrane transporter activity | Molecular function | 16 | 464 | 370 | 17548 | 1.6354147250699 | 0.0378080660179238 | 1 | 1.42241553737498 | 0.0432432432432432 | P2RX2//GRIN2B//ANO3//BEST1//CHRNA3//KCNIP3//SCN3B//SCN1A//SCN7A//LRRC38//KCNQ4//KCNIP2//SLC26A1//ABCC8//JPH1//TOMM40L// |
| GO:0035613 | RNA stem-loop binding | Molecular function | 2 | 15 | 370 | 17548 | 6.3236036036036 | 0.0388443971005432 | 1 | 1.41067161479867 | 0.00540540540540541 | SNRPA//CPEB3// |
| GO:0005178 | integrin binding | Molecular function | 6 | 118 | 370 | 17548 | 2.41154374713697 | 0.0389185623819683 | 1 | 1.40984321056991 | 0.0162162162162162 | NISCH//EMP2//ITGA5//TLN1//JAM3//FERMT3// |
| GO:0000979 | RNA polymerase II core promoter sequence-specific DNA binding | Molecular function | 3 | 36 | 370 | 17548 | 3.95225225225225 | 0.0397567586398071 | 1 | 1.40058903076942 | 0.00810810810810811 | HEYL//ZFP64//PAX8// |
| GO:0022838 | substrate-specific channel activity | Molecular function | 15 | 437 | 370 | 17548 | 1.62792998948605 | 0.0448412382586917 | 1 | 1.34832240318028 | 0.0405405405405405 | P2RX2//GRIN2B//ANO3//BEST1//CHRNA3//SCN3B//SCN1A//SCN7A//LRRC38//KCNQ4//KCNIP2//SLC26A1//KCNIP3//ABCC8//JPH1// |
| GO:0005488 | binding | Molecular function | 328 | 15006 | 370 | 17548 | 1.03665632845961 | 0.0454864910996829 | 1 | 1.34211756404544 | 0.886486486486487 | ACADL//CPLX1//LRRK2//VAMP3//GOSR2//SLC27A4//PEX5//MARK1//OXSR1//LARP1//HMGB3//BAG5//BAG2//CPEB3//PURA//CREB3L3//VEZF1//PRDM4//DLX5//ELK1//KCNIP3//SMAD1//PAX5//SKIL//PAX8//ACTL6A//PAXBP1//HEYL//ZFP64//FOXP3//BHLHE40//GNAO1//GNAZ//PROK1//TLN1//NUCB1//RIC8B//HIF1A//DPYS//KRBOX1//SPRN//ENOX1//ZNF804A//ZBTB6//ZNF618//MCMDC2//EPAS1//FOXG1//ZBTB43//CARHSP1//MLF1//POLR2E//STRBP//CREBZF//PRDM12//SIM1//ZNF124//CXXC4//CREB5//PRKAA1//RPP14//EIF4A2//EIF4G2//EWSR1//MAPRE1//RRP1B//R3HDM1//SLC25A5//PDIA3//HMGN2//NOVA1//UCHL5//RBFOX1//THUMPD1//UBFD1//PAPOLB//RTN4//PTBP1//RBM3//SNRPA//TPD52L2//U2AF1//YWHAZ//MEX3B//ZCCHC9//COPS5//MAEA//PARVA//EPS8L2//MLPH//DIXDC1//MICAL2//NOS3//SKAP1//PARD6B//RALGPS2//ARHGEF6//GRIN2B//NCAM1//TRDN//CADM2//TNFSF4//TNFSF14//NDP//SFRP1//EBI3//GDF9//BMP10//IFNK//FAM83B//ITGA5//RASL11B//NISCH//EMP2//JAM3//FERMT3//TG//FABP2//CYP4F3//CYP4F2//CH25H//AOC3//SYT14//GPD2//KCNIP2//PPP3CA//PPP3CB//PCDHB3//VLDLR//TIMM23//ACTR3//PYURF//CALCOCO2//CDKN1B//EMC8//PPIH//RALBP1//TOMM34//PNRC1//FERMT2//PTPRT//AKAP10//CHRNA3//AP1S1//SLAIN1//UBXN10//LYPD6//PACRGL//LSM11//UBXN2B//NEK7//CTH//CTNND2//USP54//SLC35G1//C18ORF54//UBXN2A//SNX31//SPIN3//FAM9A//FAM9C//DLAT//AGTR1//EFNA3//FBXO45//HIPK1//PAXIP1//FAM168A//MGRN1//ATP1B4//CAPN7//LDOC1//ARFIP2//FZD2//SULT4A1//NAT9//FBXL3//MYCBP//PTPN18//SH3YL1//GPR3//WDR91//NRBF2//CDR2L//HOXC9//MCIDAS//IGSF1//IL4R//FAM111B//KIF2A//KRT2//C16ORF87//CCDC103//TRABD2B//RHOH//CD46//MEA1//MRC1//NRAS//PALM//PLLP//RLIM//INSIG2//PMAIP1//USP53//PPP1R2//TMEM248//SHQ1//NUDT15//VPS53//RFK//DRAM1//TBC1D22B//MAP7D1//ASF1B//PRKAR2B//BRK1//KMT2E//MAPK4//KLHL7//FAM214A//GRIPAP1//PSMD7//WDFY1//SEMA4G//TP53INP2//PCTP//RPE//RRAS//BFSP1//CXCL11//CEP85//SKP1//SMPD1//STATH//HSPA13//TMPRSS2//TTK//UBE2D3//UBE2E1//YWHAB//RPAP3//FZD3//NUP214//TNKS2//NDFIP1//TXNDC5//TRIM7//CAMLG//CASP7//RNF135//OGT//CGNL1//PARG//STON2//KBTBD6//NFS1//KCNQ4//SESTD1//BUB3//CD8A//NREP//COPS2//SH3BP5//SCAMP1//CD81//PPP1R26//SEPT7//HTRA4//ACTR2//UBE4B//ABCA13//P2RX2//ABCC8//UCK2//PIP4K2C//RABL3//RHOF//FGF11//PABPC3//CHRM1//PGGT1B//ALAD//ZSWIM6//AGBL2//ADAM33//STARD4//DBI//DDAH1//ALDH1A2//EVPLL//TSPAN14//TTC28//PPP1R3F//AADAT//CLEC3A//CLEC1A//CNTN2//DNAJB12//TOMM40L//RNF169//RNF217//TNFAIP8L3//TULP4//ZNF322//CLDN18//BEST1//BEX4//LETM2//OTP//TLX2//PFDN1//LRRC38//SCN3B//PRUNE2//PPTC7//RNF149//IDI1//ME2//NPTX1//UPB1//RSBN1//SNN//ITGA8//BSN//KDELR3//ANO3//TNFRSF8// |
| GO:0019905 | syntaxin binding | Molecular function | 5 | 93 | 370 | 17548 | 2.54984016274339 | 0.046849214822326 | 1 | 1.32929768335013 | 0.0135135135135135 | CPLX1//LRRK2//VAMP3//SYT14//ABCC8// |
| GO:0015405 | P-P-bond-hydrolysis-driven transmembrane transporter activity | Molecular function | 6 | 124 | 370 | 17548 | 2.29485614646905 | 0.0476021241413091 | 1 | 1.32237366739995 | 0.0162162162162162 | ATP1B4//ATP6V1G2//TIMM23//RALBP1//ABCA13//ABCC8// |
| GO:0008320 | protein transmembrane transporter activity | Molecular function | 2 | 17 | 370 | 17548 | 5.57965023847377 | 0.0489486211426783 | 1 | 1.3102595375393 | 0.00540540540540541 | TIMM23//TOMM40L// |
| GO:0015399 | primary active transmembrane transporter activity | Molecular function | 6 | 125 | 370 | 17548 | 2.2764972972973 | 0.0491567247766197 | 1 | 1.30841706107497 | 0.0162162162162162 | ATP1B4//ATP6V1G2//TIMM23//RALBP1//ABCA13//ABCC8// |
| GO:0044877 | protein-containing complex binding | Molecular function | 27 | 916 | 370 | 17548 | 1.39795822022896 | 0.049999508401331 | 1 | 1.30103426565676 | 0.072972972972973 | IFNK//NISCH//EMP2//ITGA5//TLN1//JAM3//FERMT3//EVPLL//CD81//RNF169//HMGN2//ACTL6A//GNAO1//GNAZ//VLDLR//PPIH//RNF135//LETM2//CPEB3//ACTR3//ACTR2//UCHL5//LRRK2//ALAD//SNRPA//CDKN1B//BRK1// |
